# 979. A Cluster-randomized Controlled Trial of Enhanced Carbapenem-resistant *Enterobacteriaceae* Prevention Program at General Medicine Wards, Siriraj Hospital

**DOI:** 10.1093/ofid/ofad500.034

**Published:** 2023-11-27

**Authors:** Apiradee Taweesuk, Siriporn Rachakhom, Walaiporn Wangchinda, Visanu Thamlikitkul, Susan Assanasen, Pinyo Rattanaumpawan

**Affiliations:** Faculty of Medicine Siriraj Hospital, Mahidol University, Bangkok, Krung Thep, Thailand; Faculty of Medicine Siriraj Hospital, Mahidol University, Bangkok, Krung Thep, Thailand; Faculty of Medicine Siriraj Hospital, Mahidol University, Bangkok, Krung Thep, Thailand; Faculty of Medicine Siriraj Hospital, Mahidol University, Bangkok, Krung Thep, Thailand; Siriraj Hospital, Bankok Noi, Krung Thep, Thailand; Faculty of Medicine Siriraj Hospital, Mahidol University, Bangkok, Krung Thep, Thailand

## Abstract

**Background:**

Carbapenem-resistant *Enterobacteriaceae* (CRE) colonization is an important risk factor for CRE infection. Most of Infection prevention control (IC) strategies to reduce CRE colonization were recommended to implement in a private room setting. Data on effectiveness of IC program in general medicine wards is limited.

**Methods:**

During February-October 2021, a cluster-randomized controlled trial was conducted among high risk for CRE-adult patients at six general medical wards, Siriraj Hospital. Six wards were randomized to receive either the standard IC care (sIC) or the enhanced CRE prevention program (eIC) plus sIC. The active CRE surveillance of stool or rectal swab specimen was performed in both groups within the first 72 hours of enrollment and then every 7 days. The eIC included a monthly healthcare education and feedback session, a real-time alert of CRE-acquisition from the active surveillance, and the CRE contact precaution visual reminder. The sIC included the cohorting and contact precaution of a CRE-acquisitioned patient, and a real-time alert of CRE-acquisition from the clinical specimen.

**Results:**

There were 174 patients (with 1684 patient-days) in the intervention group and 189 patients (with 1517 patient-days) in the control group. Baseline characteristics were comparable between two groups. The cumulative incidence of CRE-acquisition was slightly lower in the intervention group (36.8% (64/174) vs. 46.6% (88/189);*p=0.059*). The incidence rate of CRE-acquisition was significantly lower in the intervention group (0.038 vs. 0.058;*p=0.007*). The median [range] of CRE-acquisition free time (6 days [1-73] vs. 5 days [1-81];*p=0.84*) were comparable. *K. pneumonia*e was the most common CRE pathogen isolated from the surveillance and clinical specimens. No difference in all-cause mortality and length of stay between the two groups was observed.

**Conclusion:**

The incidence of CRE acquisition at our institute was remarkably high. The eIC could reduce the incidence of CRE-acquisition and lengthen the CRE-acquisition free time. Future study should be done to explore the benefit on morbidity and mortality outcomes.

**Disclosures:**

**All Authors**: No reported disclosures

